# Aspiration-Based Partner Switching Boosts Cooperation in Social Dilemmas

**DOI:** 10.1371/journal.pone.0097866

**Published:** 2014-06-04

**Authors:** Zhi Li, Zhihu Yang, Te Wu, Long Wang

**Affiliations:** 1 Center for Complex Systems, Department of Automatic Control Engineering, Xidian University, Xi’an, China; 2 Center for Systems and Control, State Key Laboratory for Turbulence and Complex Systems, College of Engineering, Peking University, Beijing, China; Universidad Carlos III de Madrid, Spain

## Abstract

Most previous studies concerning linking dynamics often assumed that links pairing individuals should be identified and treated differently during topology adjusting procedure, in order to promote cooperation. A common assumption was that cooperators were expected to avoid being exploited by quickly breaking up relationships with defectors. Then the so-called prosocial links linking two cooperators (abbreviated as *CC* links hereafter) would be much favored by evolution, whereby cooperation was promoted. However, we suggest that this is not always necessary. Here, we developed a minimal model in which an aspiration-based partner switching mechanism was embedded to regulate the evolution of cooperation in social dilemmas. Individuals adjusted social ties in a self-questioning manner in line with the learning theory. Less game information was involved during dynamic linking and all links were tackled anonymously irrespective of their types (i.e., *CD* links, *DD* links, or *CC* links). The main results indicate that cooperation flourishes for a broad range of parameters. The denser the underlying network, the more difficult the evolution of cooperation. More importantly, moderate aspirations do much better in promoting the evolution of altruistic behavior and for most cases there exists the optimal aspiration level that most benefits cooperation. Too strong or too weak selection intensity turns out to be pretty conducive to the evolution of cooperation in such a dynamical system.

## Introduction

Cooperation plays a key role in the establishment and development of human civilization [1], as well as in modulating self-renewal of a precursor cell consisting of various microsystems [2]. Despite its universality and importance, the emergence and maintenance of cooperation has long since puzzled scientists of different disciplines, based on the fact that cooperation often benefits defectors (**D**) at the expense of cooperators (**C**), which, in all probability, can lead to the extinction of altruism in the competition with selfishness and thus gives rise to various social dilemmas, representative of which are the prisoner’s dilemma game (PDG) [3]–[19], the snowdrift game (SG) [20]–[24] and the public goods game (PGG) [25]–[32]. In this study, we mainly focus on the PDG and SG. In such two-person games, two individuals can simultaneously choose to cooperate or to defect. They will receive reward payoff 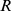 for mutual cooperation and punishment payoff 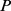 for mutual defection, respectively. If one cooperates and the other defects, then the cooperator obtains the sucker’s payoff 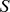 while the defector gets the highest payoff 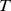. These payoff parameters meet 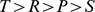 for the PDG, and 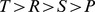 for the SG. According to such a ranking, one can easily figure out that the best respond for an individual in the PDG is to defect, irrespective of the strategy of the opponent, and also that the optimal choice for an individual in the SG depends on the other’s behavior: to defect when the other cooperates or to cooperate if the other defects. However, no matter in which dilemma, the best solution for the group is always mutual cooperation. No doubt, it creates an irreconcilable conflict between what is the best for the individual and what is the best for the group.

Aimed at solving these dilemmas, a great number of approaches have been proposed in the context of evolutionary game theory and many notable achievements have been made over the past few decades [33]–[38]. Among the techniques designed to support cooperation, linking dynamics has attracted considerable attentions [39], [40]. What’s more, some researchers find that dynamical networks perform much better than static graphs in terms of boosting the evolution of cooperative behavior under certain circumstances [41]–[47]. For more detailed information, please refer to the review [48]. In these studies, links can often be identified and be explicitly or implicitly classified into three types such as *CC*, *CD*, *DD* links, according to the identities of their endpoints. Then different dynamics occurs with respect to different types of connections during the topology restructuring stage. Generally, the prosocial *CC* links are apt to be preserved, whereas the undesirable *CD* and *DD* links are more prone to being severed. In this way, corresponding schemes can often help enhance reciprocal interactions between cooperators whereby cooperators can be protected against nasty exploitation and fierce invasions by defectors, and finally offers cooperation a promising evolutionary fate.

In spite of their remarkable achievements in solving the concerned social dilemmas, these studies, in the view of most researchers, may still have some imperfect aspects that need to be further improved. On the one hand, the type-dependent bias toward *CC* links as well as the type-dependent privilege for cooperators, frequently employed by some authors, has always involved many details about the information of current strategies of the population. This, together with the discrimination against defectors in the partner selection process, seems a little rigorous or somewhat unreasonable. To avoid these seemingly rigid requirements, we have studied the effects of adaptive dynamical linking on the evolution of cooperation, and found that endowing individuals with the ability to adaptively adjust the duration of social ties based on the performance of their rivals can greatly ease social dilemmas in the networked populations [49]. More significantly, our main results have implied that the rationality and selfishness of a single agent in adjusting social ties can contribute to the progress of altruism of the whole population.

On the other hand, learning theory suggests that individuals can often adopt a self-questioning mechanism to modulate their behavior, e.g., to change behavior based only on personal aspirations or expectations, rather than on other evaluation criterions [50]–[58]. Therefore, it is natural and also interesting to consider other incentives regarding partner switching within the framework of evolutionary game theory with structured populations. Inspired by this, here via taking above two factors into account we propose an aspiration-based partner switching scheme to check its influences on the evolution of cooperation, without tracking link types as well as identities of game players. For simplicity, the aspiration considered in this model just characterizes the average level of tolerance or dissatisfaction of the population as in [57], [58] and it does not differ from agent to agent or change over time.

## Results

**Figure 1:**
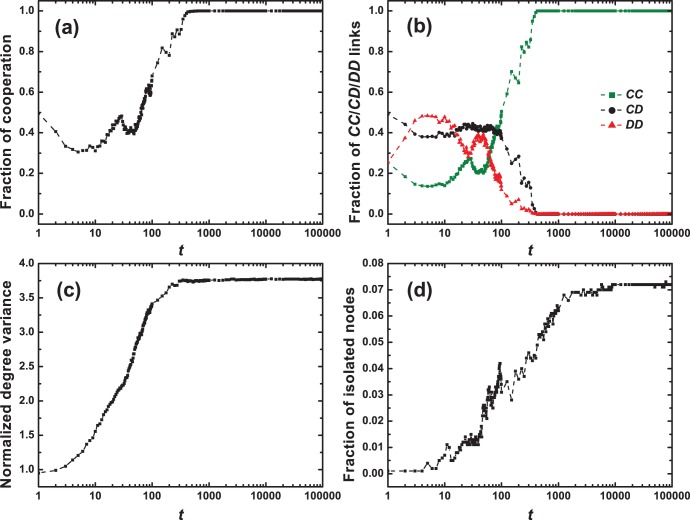
Entangled dynamics of strategies and graphs in the PDG. (a) fraction of cooperation, (b) fraction of *CC*/*CD*/*DD* links, (c) normalized degree variance 
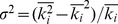
, (d) fraction of isolated nodes. Here, individuals adaptively adjust social ties in a self-questioning way based on the learning theory, and less information concerning strategies is involved during the partner switching process. Even so, this simple and smart mechanism successfully drives the system to the full cooperation state without spying on the types of links as well as the types of individuals. Initially, each individual is randomly designated as a cooperator or a defector with equal probability and all individuals are uniformly distributed in the network. Other parameters: 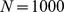, 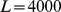, 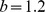, 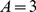, 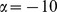, 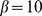, 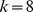.

**Figure 2:**
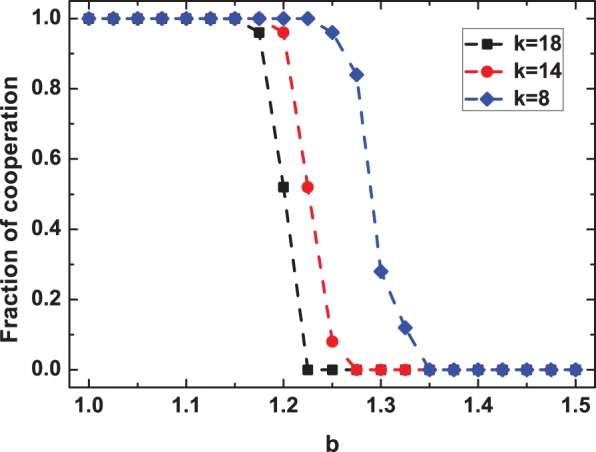
The denser the interacting network, the harder the spread of cooperation. The results are collected in the PDG. 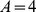. Other parameters and conditions are the same as in Fig. 1.

We first check the typical time evolution of coupled dynamics of strategies and topologies in the PDG (see Fig. 1). In such a dynamical system, prosocial *CC* links tend to be frequently replicated and to propagate within the population under the influence of aspiration-based partner switching mechanism, while unfavorable *CD*/*DD* links have been greatly suppressed as shown in Fig. 1(b). As a result, cooperation eventually wins the evolutionary race in the competition with defection. And the evolutionary dynamics can also drive the system to the full cooperation state with respect to proper system parameters as depicted in Fig. 1(a). Of great interest is that in such scenarios the normalized degree variance, often utilized by researchers [41] to track how the structure of networks transforms, has been found to climb monotonously along with the proceeding of the evolution. It implies that the underlying network is evolving toward a more heterogeneous topology, departing significantly from the initial network configuration that is subject to the Poisson degree distribution (i.e., 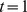, 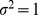 in Fig. 1(c)). Compared to initial graphs, the emergent networks witness a significant rise in the number of isolated nodes owing to linking dynamics, which is also an important factor fluctuating the heterogeneity of the resulting networks. In addition, by checking the effects of average degree 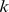 on cooperation in Fig. 2, we find that the denser the network, the harder the spread of cooperation.

**Figure 3:**
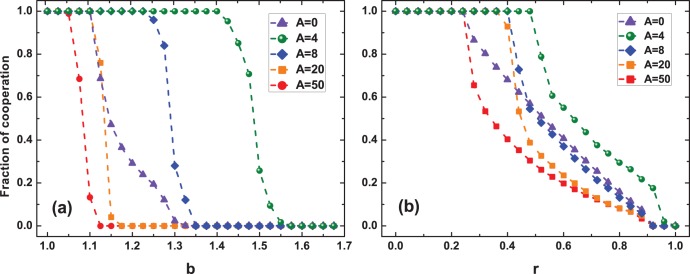
Effects of different aspiration thresholds on the evolution of cooperation in the PDG (a) and SG (b). 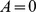
 corresponds to the results obtained on static networks. Based on this plot, one can conclude that the aspiration level plays a crucial role in the evolution of cooperation. Moderate aspirations are more efficient in terms of boosting cooperation. 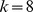 for the PDG (a), and for the SG 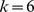 (b). Other parameters and conditions are the same as in Fig. 1.

Next, we proceed with deciphering the performance of aspiration-based partner switching in boosting the evolution of cooperative behavior in Fig. 3. As it shows, aspiration-based parter switching, in line with a self-questioning mechanism, can strikingly promote cooperation for a wide range of parameters both in the PDG (see Fig. 3(a)) and in the SG (see Fig. 3(b)), in the absence of any type-dependent biases or type-dependent privilege that may remarkably favor cooperation. This promotion effect shows a very close correlation with the aspiration level of the population (i.e., the value of 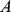). By comparison, one can figure out that intermediate aspirations perform much better in upholding cooperation, while too large values of 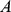 instead heavily inhibit cooperation. In fact, 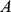 mainly affects topology dynamics from two aspects in such a dynamical model. First, 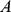 itself, as the key payoff threshold, plays an important role in determining in which case linking dynamics has the probability to happen. Aside from this, it also exerts an immense impact on the reconnecting probability 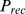 (refer to the definition of 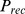 in the Model section), by which the rewiring event actually occurs. In this sense, 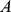 indeed corresponds to topology regulating rate, and large 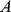 generally means frequent adjustments to social ties, while small 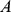 implies infrequent modulations. Thus, it is not hard to understand why in most cases intermediate aspirations are more beneficial to the evolution of cooperation, indicating that moderate adjusting rates are needed for cooperation to evolve in such a dynamic system. From here on, unless otherwise stated, all of the following analysis is based on the PDG.

**Figure 4:**
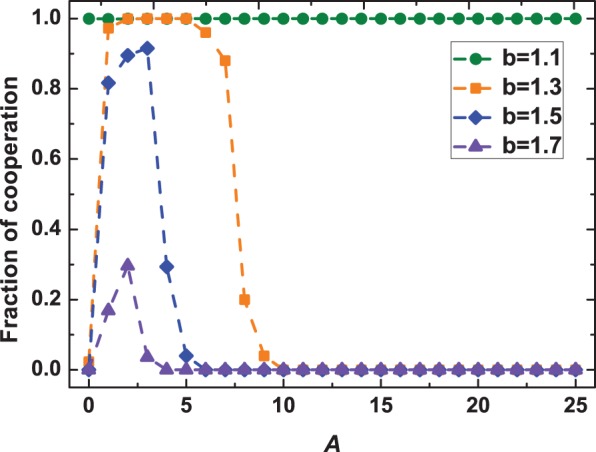
Fraction of cooperation in dependence on 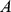 for various temptation to defect 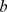 in the PDG. 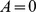
 corresponds to the results obtained on static networks. It reflects that the influences of 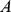 on cooperation relay closely on the challenge that cooperation is facing. For most values of 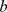, there exists the optimal 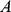 that most fosters cooperation. Other parameters and conditions are the same as in Fig. 1.

To further explore the impacts of 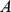, we also plot in detail the cooperation level as a function of 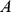 for representative values of 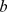 in Fig. 4. As shown in this plot, the effects of 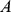 on cooperation relay closely on the strength of the challenge that cooperation is confronted with. On the one hand, for small values of 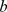, the final cooperation level in the equilibrium state nearly reveals no differences with respect to different 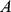. At first glance, this observation somewhat makes the intervention from aspiration-based partner switching mechanism noteless in such scenarios. However, with the growth of 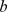, moderate or relatively small values of 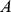 immediately show their significant advantages in upholding the evolution of altruistic behavior. Especially with respect to the case 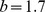, a quite hostile environment to cooperation, it depicts that cooperation can still survive with the aid of the concerned mechanism, highlighting the high efficiency of aspiration-based partner switching in facilitating cooperation. Besides, what should be further emphasized is that for most values of 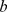, there exists the optimal aspiration level that most favors the propagation of cooperation, which is in good agreement with what we have observed in Fig. 3.

**Figure 5:**
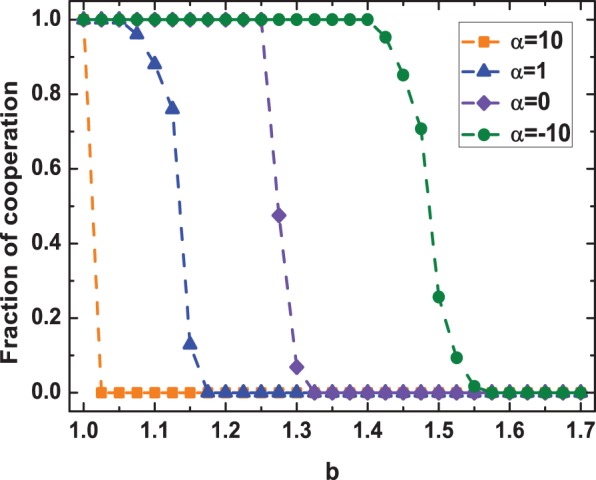
Influences of the preference in partner selection on the evolution of cooperation in the PDG. 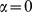
 corresponds to random partner selection in determining which one to dismiss. The plot indicates that rewiring connections with the poorer partners can often benefit the evolution of cooperation. And the smaller the value of 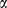, the stronger the promotion of cooperation. 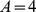. Other parameters and conditions are the same as in Fig. 1.

Typically, when individuals construct and maintain partner networks, they may have diverse preferences in considering which link to terminate. And these preferences, shown in network organizing, usually play a decisive role in determining whether cooperation can evolve. For example, Fu *et al* found that cooperation can always be competitive throughout the evolution when individuals are assumed to often terminate interactions with partners who have the lowest reputation [45]. Based on its importance, in what follows, we proceed with examining the effects of 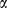 on cooperation in Fig. 5. Quite intuitively, corresponding observations demonstrate that, negative 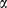 can dramatically enhance cooperation. With respect to positive 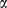, however, cooperation can often be severely damaged. And the smaller the value of 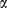, the better the promotion of cooperation. This illustrates that frequently rewiring the links with the poorer partners can be more beneficial to the spreading of cooperation, provided that individuals are being situated in unsatisfactory circumstances, and vice versa. Actually, all of these observations are in good accordance with the results reported by previous studies [59], [60], in which seeking interactions with successful or wealthy encounters has been proved to be an efficient choice for the promotion of cooperation. As a special case, the results for random partner selection (i.e., the case for 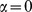) show that even a randomly and dynamically organized network can outperform a fixed topology in terms of stimulating cooperation, which no doubt, from another aspect, emphasizes the superiority of dynamical graphs in supporting the propagation of altruistic behavior.

**Figure 6:**
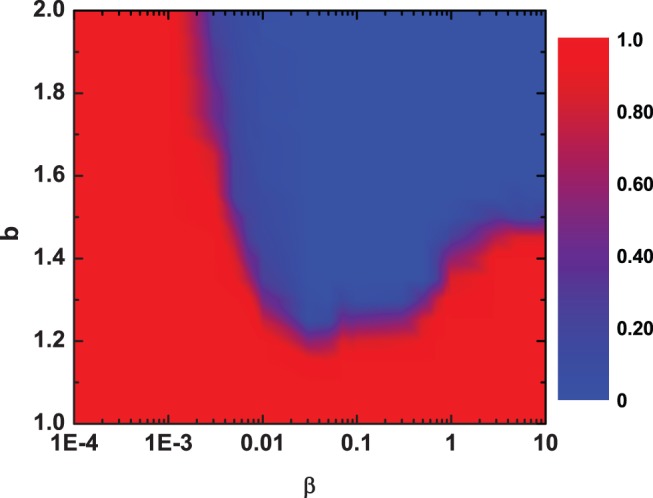
Effects of selection strength on the evolution of cooperation in the PDG. The contour of the cooperation level in dependence on 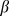 and 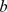 is plotted in this figure. It shows that cooperation thrives under either very strong or very weak selection strength. Particularly, for 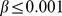 the full cooperation state can be kept in the whole range of 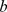. Moreover, the approximate U-shaped curve implies that there exists the worst 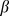 that extremely inhibits the evolution of altruism. 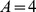. Other parameters and conditions are the same as in Fig. 1.

To the best of our knowledge, selection is usually as important as the mechanisms that are carefully designed for the promotion of cooperation in the frequency-dependent evolutionary systems. Based on its significance, we finally study the influence of selection intensity in such a dynamical environment. For this, the final cooperation level in the equilibrium state as a function of 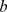 and 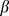 is plotted in Fig. 6, and some intriguing phenomenon is observed. First, the promotion effect of our partner switching mechanism on cooperation is quickly and heavily weakened with the slight increase of 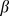 (check 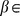
[0.001, 0.1]). Then, after undergoing a decaying ditch, this effect, to some extent, recovers again and resides in an intermediate level eventually. Thus, one can conclude that either too strong or too weak selection intensity can immensely benefit the survival of cooperation compared to the others. Especially for very weak selection intensities, the full cooperation state can be maintained in the whole range of 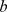 (check the results for 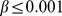 in Fig. 6). Aside from this, this approximate U-shaped curve also predict that there exists the worst 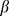 that may remarkably deter cooperation.

## Discussion

We constitute a minimal model in which aspiration-based partner switching mechanism is introduced. Each individual adaptively reshapes partner networks merely based on the relationship between their aspirations and actual payoff. We find that social dilemmas can be greatly relieved by such a self-questioning coevolutionary rule in the context of spatial games. Specifically, moderate aspirations are much better than others in boosting cooperation. And for most intermediate and relatively high temptation to defect, there often exists the optimal aspiration level that most favors altruism. Compared to most previous studies, this work has essentially evaded the extra biases or preferences that might often be imposed by some authors [41], [44], [46], [47] on cooperation or on so-called prosocial links (*CC*) in the topology adjusting stage. Then we argue that spying on the details of the current strategy configurations seems not always necessary for the evolutionary dynamics to successfully promote the evolution of cooperation. In such a dynamical system, selection intensity also has a nontrivial effect on the evolution of cooperation.

Based on the fact that diversified or personalized aspirations are ubiquitous in the real world. Then it will be more constructive and meaningful for us to further explore the scenarios where diversified or personalized aspirations are embedded in our coming work. As an outlook for future research, here we simply make a discussion about the situation where cooperators and defectors may have different aspirations. In this case, cooperators and defectors generally have different topology adjusting rates and, accordingly, the problem may be more complicated. In view of the fact that high aspirations constantly correspond to fast or frequent adjustments to underlying networks, then the general conclusion that too high aspirations always damage cooperation may remain valid in this scenario. In addition, as most previous studies show [41], [42], [44]–[46], linking dynamics benefits altruism mainly by cutting off the connection between a cooperator and a defector, and allows the cooperator to seek for another potential altruistic partner, whereby cooperation can be enhanced. However, defectors’ too much adjusting to graphs, as we know, can often aggravate their exploitation of cooperators and offset this promotion effect. As a result, the emergence, maintenance, and promotion of cooperation may potentially face a great challenge when defectors’ aspirations are far higher than that of cooperators. Our observations may help shed new light on evolutionary dynamics of dynamic systems.

## Methods

A brief introduction to our co-evolutionary game model is presented in this section. The vertices of the graph denote the individuals and the links describe the partnership between them. We carry out our studies on an Erdös-Rényi like random graph [61]. To do this, we use 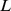 (the number of links and 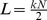) links to pair 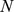 individuals at random. Statistically, each individual has the same number of partners and the whole network has an average degree 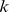. Initially, half among these 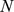 individuals are designated to be cooperators and the rest defectors with equal probability, and all of them are uniformly distributed in the entire network. In addition, each one is also endowed with a constant aspiration 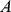, representing the average expected payoff of the population.

In each time step, all individuals (excluding isolated individuals) simultaneously interact with their directly connected neighbors and accumulate payoff. The payoff of individual 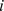 can be calculated according to Eq. (1):
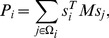
(1)where 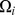 denotes neighborhood set of 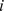 and 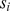 the strategy vector of 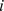. The 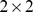 payoff matrix 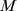 can be rescaled as Eq. (2) for the PDG, a weakened version utilized in [3]



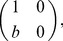
(2)and as Eq. (3) for the SG [20]

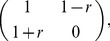
(3)where 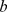 and 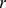 are inherent parameters of the PDG and SG, respectively, quantifying the temptation to defect in the corresponding dilemma.

Then each individual has an opportunity to update strategy through imitating the behavior of their neighbors. For example, individual 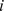 can switch her strategy by randomly selecting one, namely, 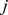, from her neighborhoods and adopting the latter’s strategy with a probability given by Fermi function as in [52]:
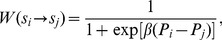
(4)and 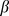 represents selection intensity (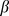 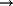
0 leads to random drift while 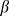 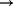 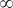 the deterministic dynamics). As for isolated individuals, we here assume that their strategies will remain unchanged in each time step.

Finally, each individual (excluding isolated ones) decides whether or not to adjust the partner network depending on the relationship between their actual payoff and the expected payoff 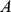. Specifically, if 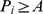, 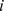 will be satisfied with the current environments and so nothing happens to the structure of the underlying network in this case. Whereas, if 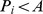, 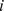 is assumed to be dissatisfied with the current configuration of the interaction network and is going to make adjustments to social ties with the probability 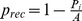. Based on this definition of 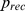, the higher the payoff 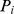, the smaller the reconnecting probability 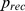. Aspiration 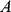 provides a global benchmark of the average tolerance or dissatisfaction of the population in determining whether or not to adjust some social ties. When the reconnecting event happens, 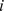 will pick out one from links belonging to her to sever and reconnect this link to another one randomly chosen from the population (excluding 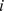
’s neighbors). Then which link will be selected to be rewired is determined by the probability.
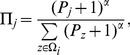
(5)where 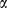 characterizes the preference of the focal individual in partner selection. Intuitively, positive 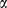 means that the connections with wealthy partners will be frequently dismissed, and vice versa. As a special case, 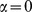 refers to random partner selection. For this case, stochasticity will be introduced into reorganizing process of interacting networks. It should be noted that the inequality 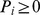 holds for any 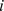, then partner switching will never happen for 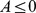 and thus we will mainly focus on non-negative values of 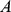 in this study.

We stop our simulations after a sufficiently long transition time (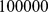 full time steps). The final level of cooperation in the equilibrium state is obtained by averaging over 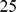 independent runs.
